# Role of telerehabilitation in the rehabilitation of children with cerebral palsy during COVID-19: A review

**DOI:** 10.1097/MD.0000000000037214

**Published:** 2024-03-01

**Authors:** Muhammad Kashif, Abdulaziz Albalwi, Syed Abid Mehdi Kazmi, Ahmad A. Alharbi, Kiran Bashir, Muhammad Aqeel Aslam, Tamjeed Ghaffar

**Affiliations:** a Department of Physical Therapy, Riphah College of Rehabilitation and Allied Health Sciences, Riphah International University, Islamabad, Pakistan; b Faculty of Applied Medical Sciences, Department of Health Rehabilitation Sciences, University of Tabuk, Tabuk, Saudi Arabia; c Department of Clinical Services, Ziauddin Group of Hospitals, Ziauddin University & Hospitals, Karachi, Pakistan; d Department of Physical Therapy, Margalla Institute of Health Sciences, Islamabad, Pakistan; e Department of Oral Medicine, Muhammad Dental College, Mirpur Khas, Pakistan; f Faculty of Medical Sciences, Department of Physical Therapy, Government College University, Faisalabad, Pakistan.

**Keywords:** cerebral palsy, coronavirus, COVID-19, pandemic, telerehabilitation, teletherapy

## Abstract

Individuals with cerebral palsy (CP) have limited mobility and are unable to actively participate in tasks that are part of their daily living. Thus, continuous therapeutic sessions are required to keep such individuals active and engaged in the environment. Due to the coronavirus disease of 2019 (COVID-19) lockdowns, rehabilitation for children with CP was inhibited which consequently put them at risk of losing their functional gains which were obtained through previous in-person therapies. In order to avoid this, an alternate to conventional therapy was required and this rendered it necessary to review the role of telerehabilitation (TR) and its various modes for the rehabilitation of children with CP. This study aimed to explore the effectiveness of TR for children with CP during COVID-19 through the present literature and to determine if TR is an alternate to conventional physical therapy in children with CP during the coronavirus outbreak. This scoping review was conducted by searching different databases such as PubMed, Cochrane Collaboration, Medline, and Google Scholar on the basis of inclusion criteria. Screening was performed from January 2019 to June 2022 and the initial screening attempt returned 469 studies. After applying the aforementioned criteria, all impertinent studies were excluded which resulted in 28 studies being included for this review as they contained information about the effectiveness of TR on children with CP during COVID-19. These 28 articles included randomised controlled trials, surveys, reviews, clinical trials, case reports, prospective studies, editorials, and longitudinal studies. Three out of the 7 randomised controlled trial studies revealed that action observation treatment can be a useful approach for TR in child with CP during similar pandemics. The other 3 studies supported the use of computer-based games, robots, nonimmersive virtual reality, and wearable haptic devices as a significant means of TR in child with CP as an alternate to routine therapy during COVID-19. TR is an affable mode of rehabilitation specifically for the pediatric population. In the future, it can be an alternate to routine therapy for those who are unlikely to get daily access to in-person therapeutic sessions due to various reasons or circumstances.

## 1. Introduction

Poor mobility and irregular posture are the main attributes of cerebral palsy (CP). It is considered to be a neurodevelopment condition that may make it difficult to learn, remember, or apply specific skills or sets of knowledge.^[1]^ Common symptoms that are found in children with CP include deficiencies in sensation, rigidity, stiffness, and myasthenia that is, weakness in muscles. Similarly, children with CP also frequently experience functional deprivation and heavily rely on others for a variety of daily living tasks and activities as a result of these critical symptoms.^[2]^ To this extent, children suffering from CP are more prone and susceptible to the development of issues that are linked with the musculoskeletal system. These musculoskeletal issues are chiefly associated with poor mobility, imbalance, deprivation of selective motor tasks, as well as fluctuations in the normal tone of muscles and physical growth among children.^[3]^ Furthermore, the widespread cause of movement impairment is CP based on previous studies, the global prevalence is 2 to 3 per 1000 live births.^[4–7]^

Depending on their level of functioning, children with CP may face a wide range of challenges in fulfilling their ecological and environmental requirements on a daily basis. Some minors with CP, for example, may present with postural and walking issues, while others may struggle to accomplish academic chores. Nonetheless, others may not be able to effectively articulate their requirements and may depend frequently on their parents and caregivers for support.^[8–10]^ Such circumstances may be deemed extremely harmful, and they could have a significant impact on their quality of life.^[11–13]^

Given these challenges, it is essential that people with CP have uninterrupted access to rehabilitation and therapy.^[14]^ The COVID-19 pandemic occurrences caused by the coronavirus disease had a significant impact on human life.^[15]^ In order to prevent the stretch of the coronavirus disease (COVID-19) pandemic effectively, governments around the world implemented extreme restrictions such as maintaining a 6 feet social distance, forced quarantines, lockdowns, and the closures of schools^[16]^ Moreover, the situation is exacerbated by the increased load placed on families, mentors and caregivers due to lockdowns, the delay of classes at school, and the hindrances in rehabilitation programs such as sessions for vocalization, physical therapy (PT), and incentive trainings.^[15]^

Due to the direct effects of the COVID-19 pandemic, social isolation has emerged as a result of social distancing, putting children in particular, at risk of developing relational discomfort. Many minors were compelled to stay at home due to the emergence of the aforementioned coronavirus (COVID-19).^[17]^ In essence, children with CP were confined to their homes and could no longer access the aforesaid treatments, resulting in unfavorable clinical and health outcomes.^[18]^ Evidently, there has been an increase by medical practitioners in the use of telerehabilitation (TR) that is, rehabilitation in which electronic methods are being used to exchange medical information among people from different geographical regions. Additionally, World Confederation for PT also encouraged the use of home-based rehabilitation or telehealth in order to promote easy access to healthcare and rehabilitation therapists. This was to allow for the new approach to be promoted as a result of this shifting environment of medical practice.^[19]^ In light of the aforementioned literature, the purpose of this scoping review was to present extensive information on the use of TR for managing CP during the COVID-19 and lockdown periods. This review is timely given that TR interventions are needed and being developed in the wake of the COVID-19 pandemic. Moreover, this review will contribute to the current evidence base as despite 3 previous reviews having been conducted in this area, 1 was not specific to children with CP,^[20]^ and 1 only focused on therapy for the upper extremities,^[21]^ and 1 review was conducted during COVID-19 which reported studies primarily regarding telerehabilitation before the COVID-19 pandemic occurred.^[22]^

## 2. Methodology

### 2.1. Search strategy

This scoping review was conducted by searching different databases such as PubMed, Cochrane Collaboration, Medline, and Google Scholar on the basis of specified inclusion criteria. Screening was performed from January 2020 to June 2022. Search terms included telemedicine, teletherapy, TR, home-based rehabilitation, CP, unilateral CP, pandemic, coronavirus, COVID-19, as well as quarantine.

### 2.2. Inclusion and exclusion criteria

Research papers that were published in English during COVID-19, reported the effect of TR on children with CP were included in the systematic review. Research papers that involved populations without CP but with other neurological disorders were excluded from the study. Similarly, unpublished literature and research papers of which the full text was not available were omitted.

### 2.3. Study selection and data extraction

**Figure 1.**
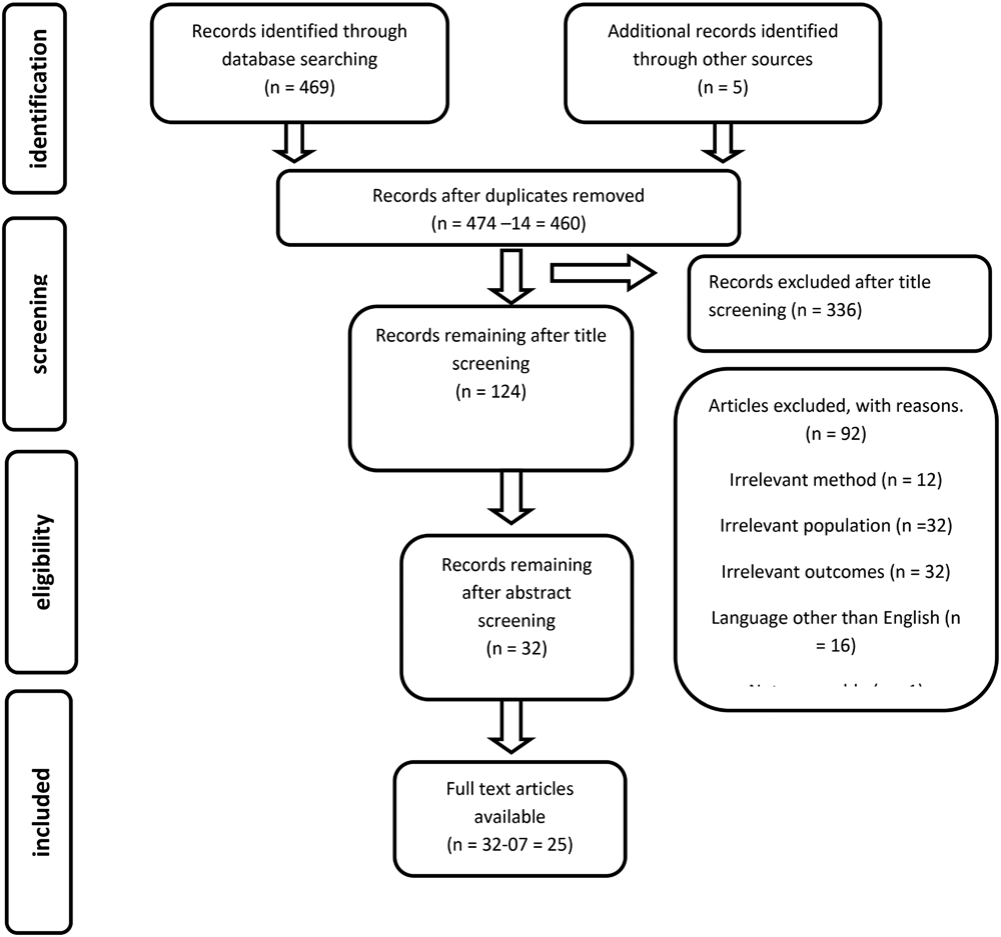
Flowchart of including studies.

Database searches identified 469 records initially and additional sources identified 5 records, that is, backward reference searches were conducted on all papers identified for inclusion through the previous search strategies. From the findings, 14 records were removed due to duplication and a total of 124 records remained after a title screening. Ninety-two articles were excluded, with reasons. Hence, 25 research studies were included in this scoping review (Fig. 1).

### 2.4. Data extraction

Data were abstracted from those full articles that met our inclusion criteria. We developed a data charting form to record the following information for each study: author, year of publication, study design, country, age, sample size, and results/conclusions. The scoping review methodology does not demand the critical evaluation of selected articles. Full-text articles whose inclusion was deemed inconclusive were identified and discussed, and all full-text articles’ references were checked for additional relevant citations.

## 3. Results

In this scoping review, we screened 469 journal articles and included 25 articles that contained information about the effectiveness of TR on children with CP during COVID-19. These 25 articles included surveys, mini-reviews, clinical trials, case reports, prospective studies, editorials, systematic reviews, research reports, point of view studies.

Based on the results of the clinical trial studies included in this study, action observation,^[23]^ computer-based games,^[24]^ robots, nonimmersive virtual reality,^[24,25]^ wearable haptic devices,^[25]^ upper limb functions of children with hemiplegic CP^[26]^ and modified constraint-induced movement therapy^[26]^ and can be used as a significant means of TR for children with CP and as an alternative to routine therapy during COVID-19 (Table 1).

**Table 1:** Data extraction.

Sr. no	Author’s name	Year	Study type	Country	Age of the participants	Sample size	Results/conclusion
1	Stasolla and Ciarmoli^[27]^	2020	A mini-review	Italy	N/A	7 studies with 333 participants	TR’s usability and validity in children with CP were demonstrated by data. Their quality of life was much improved, and carers’ burden was significantly reduced.
2	Molinaro et al^[23]^	2020	A pilot study	Italy	5–12 yr	10 children	Action observation treatment is a viable strategy that can be employed in a telerehabilitation setting on a broad scale.
3	Fazzi and Galli^[28]^	2020	Letter to the editor	Italy	N/A	N/A	After COVID-19, the adoption of modern communication technology during the pandemic could become a useful tool in daily clinical practice and treatment of children with neuro disorder.
4	Ben-Pazi et al^[29]^	2020	A mini-review	Israel	N/A	N/A	The authors of this mini review speculate that the growth of telemedicine programs could facilitate effective workflows in the treatment of individuals with cerebral palsy, proposing a platform called “Guide-lined and Structured Continuous Care” (TGSCC).
5	Demers et al^[24]^	2020	Perspective	USA	N/A	N/A	Children with disabilities, including those with cerebral palsy, can be effectively rehabilitated at home using active video games and low-cost virtual reality, either synchronously or asynchronously, in conjunction with a therapist, in the event of a global pandemic. In the event of a global pandemic, it suggests ways to incorporate active video games and low-cost virtual reality into clinical practice.
6	Bortone et al^[25]^	2020	Cross-over design	Italy	Mean age = 10.13 ± 2.59 yr old	8 CP children Allocated to VR assisted intervention: 04 Allocated to conventional intervention: 04	This study found that immersive VR and wearable haptic devices can improve upper extremity function in children with neuromotor impairments including CP compared to conventional therapy.
7	Rezk et al^[26]^	2020	A randomized controlled trial	Egypt	TR group = 6.23 control group = 6.17	Control group (N = 15) TR group (N = 15)	Telerehabilitation employing home-based modified constraint-induced movement therapy can be a useful treatment option for improving upper limb function in children with hemiplegic cerebral palsy, particularly during epidemic spread and the restrictions that come with it.
8	Rao^[30]^	2020	Point of view	India	N/A	N/A	As a result of COVID-19, PT assessment and rehabilitation of children with disabilities need to be reevaluated and revamped. This is done by shifting our focus toward home-based and family-centered care. It is possible to bridge the gap in PT services delivery through such approaches as telerehabilitation.
9	Maitre et al^[31]^	2021	Implementation study		N/A	N/A	Telehealth was used for neurodevelopmental surveillance in a tertiary high-risk infant follow-up clinic; broader applications of telehealth could overcome access obstacles.
10	Micheletti et al^[32]^	2021	Longitudinal study	Italy	63 mo	Nine pre-school children	The finding of the study reported that in children with neuromotor and intellectual impairments, an intensive, parent-centered, telepractice-based intervention has the potential to improve lexical and syntactic task scores.
11	Amirthalingam et al^[33]^	2021	A systematic review	USA	N/A	13 articles	Incorporating virtual reality into therapy has a positive impact on motor function recovery, particularly in the upper and lower limbs for balance, walking, and posture in children with CP.
12	Khan^[34]^	2021	Editorial	Pakistan	N/A	N/A	Telerehabilitation is said to be a great alternative to facility-based rehabilitation institutions that is both cost-efficient and effective.
13	Asano et al^[35]^	2021	A case report	Japan	7 yr	1 boy	After commencing telemedicine PT, the patient’s condition improved as a result of rehabilitation.
14	Sarti et al^[17]^	2021	Clinical trial	Italy	N/A	56 children, 20 CP child, and 36 children with special learning disabilities	In comparison to the other 2 groups, children who received telerehabilitation had the greatest results on the learning scale.
15	Chen et al^[36]^	2021	Clinical trial	Taiwan	5–12-yr-old children	In phase 1, 10 children in phase 2, 8 children	The program’s design was CIT-specific and motivational for children with unilateral CP, according to phase 1 results. In the 5th week of the intervention, game performance and BBT scores began to show stable gains in phase 2.
16	Assenza et al^[37]^	2021	Survey study	Italy	N/A	362 patients (270 children and 92 adults)	A medium-high degree of perception and a high level of satisfaction were stated by all 144 caregivers, 25 adult patients, and 50 experts.
17	Silva et al^[38]^	2021	Survey pilot study	UK	Mean age = 19 yr	44 individuals	The most notable finding in this cross-sectional study was that both groups experienced a significant. Increase in perceived exertion (as measured by the Borg scale) throughout practice, with CP having a higher rating of felt exertion.
18	Alonazi^[39]^	2021	Systematic review	Saudi Arabia	N/A	13 articles included	In children with various conditions during COVID-19 Overall, all of the research found that telerehabilitation has a good impact on PT.
19	Sobrepera et al^[40]^	2021	Survey study	USA	40.4 yr	423 participants	Therapists who are interested in adopting Lil’Flo have a considerably higher perception of its usefulness across all usefulness parameters.
20	Tamboosi et al^[41]^	2021	A narrative review	Saudi Arabia	N/A	5 studies	All research included in the evaluation of telerehabilitation for children with UCP is supported.
21	Chen et al^[42]^	2021	Pediatric rehabilitation perspective	New York	N/A	N/A	It is becoming increasingly important in pediatric rehabilitation to use telemedicine as a safe and effective means of communication, evaluation, and treatment, especially amid the current COVID-19 outbreak.
22	Akulwar-Tajane and Bhatt^[43]^	2021	Prospective quasi-experimental	India	3.02 ± 2.1	21 children	Clinical results for children have improved as a result of telerehabilitation. Families expressed a desire for in-person sessions despite their happiness with TR services.
23	Nuara et al^[44]^	2022	A review	Italy	N/A	N/A	It is likely that telerehabilitation has many advantages that go well beyond its physical distancing effects, alleviating the criticalities of daily neuro-rehabilitative practice, and paving the way for the vision of mixed care models, in which hospital-based procedures and telerehabilitative ones are complementary.
24	Karatekin et al^[45]^	2021	Research report	Turkey	N/A	110 children with CP	Telemedicine should be used to follow cerebral palsy children at short intervals, and in the hospital when necessary.
25	Asano et al^[35]^	2021	A case report	Japan	N/A	A 7-yr-old boy with CP	It is crucial to maintain motor function in children with CP who are nonambulatory through frequent PT. Telerehabilitation must be developed urgently as a future countermeasure.

BBT = box and block test, CIT = constrain induced therapy, COVID-19 = coronavirus disease of 2019, CP = cerebral palsy, N/A = not applicable, PT = physical therapy, TR = telerehabilitation, UCP = unilateral cerebral palsy, VR = virtual reality.

A systematic review included in this review examined studies in which telerehabilitation was provided to children suffering from different conditions such as autism spectrum disorders, CP and other neuromuscular diseases,^[44]^ systematic reviews included studies showing the usefulness of telerehabilitation in children with CP as well as in patients with stroke tendencies and determined that TR can benefit stroke patients and children with CP, especially for the recovery of upper limbs in unilateral CP,^[39]^ 1 narrative review investigated the effectiveness of TR for children diagnosed with unilateral CP.^[41]^ Moreover, both the mini-reviews demonstrated the usability and validity of TR in children with CP. It was clear that the quality of life in the child with CP was significantly improved, and the caregiver’s burden was reduced. It must also be noted that patients preferred visual teleconsultations rather than phone or e-mail consultations^[27,29]^ (Table 1). Moreover, the results of the study confirmed that telerehabilitation is a safe means of communication^[45]^ and families of CP children are satisfied with it,^[37]^ and it should be developed as a countermeasure in the context of COVID-19 pandemic^[35]^ (Table 1).

## 4. Discussion

The primary intention of this scoping review was to report the effectiveness of TR for the management of child CP during COVID-19. As per author’s knowledge, there had been no reviews that determined the use of TR in the management of children with CP during COVID-19. All the articles included in the review reported that TR plays a significant role in the management of children with CP during COVID-19. The studies supported the use of action observation treatment (AOT), nonimmersive virtual reality, computer-based games etc, as a useful mode of TR.

### 4.1. AOT for management of CP

During COVID-19, action observation was found to be an important component of rehabilitation of CP while staying at home as a part of telerehabilitation. AOT is a relatively new intervention in PT that comprises of inspecting meaningful tasks and conducting them with the purpose to replicate afterward.^[46,47]^ It is mostly explained as when an individual is requested to monitor behavior displayed via a movie clip rather conducted by a controller with a purpose to portray, attempt, and successfully implement them after inspection. As a consequence of monitoring activities, mirror neuron mechanisms may operate tasks of upper extremities by basically conducting related neural structures.^[48,49]^ According to the existing data, AOT is the most recent therapeutic method along with other treatments available for pediatric population diagnosed with unilateral CP on functional capacity of upper extremity and activities of daily living.^[50]^ AOT has also been used to help children with CP to restore the motor activities of upper extremities. Many studies demonstrated the viability of AOT for enhancing motor tasks of upper extremities among pediatric patients diagnosed with CP.^[51–53]^

### 4.2. Computer-based games and nonimmersive virtual reality as a mode of telerehabilitation for management of CP

The findings of the current review concluded that computer-based games and virtual reality can be an effective method of introducing TR during COVID-19 for CP management. A web-based E-game using the Wii Fit Balance Board reveals that rehabilitative tasks integrated among children with CP to enhance their stability during static and dynamic position, was secure, engaging, and viable for domestic purposes.^[54]^ The conventional rehabilitation exercises overlook these compensatory movements, however, digital games that give feedback on accommodative movements are advantageous for stability and balance training sessions in a domestic environment where healthcare practitioners are unable to direct movements or give verbal feedback on quality of movement.^[54]^ Moreover, with the increasing availability of desktop-dependent systems, therapeutic interventions are enormously utilizing virtual reality (VR) surroundings to improve activity training. Furthermore, VR has presently been investigated as a potential approach to enhance functional ability and movement in pediatrics diagnosed with CP.^[55,56]^ In light of the available research, VR is beneficial as it can easily be exercised in a domestic environment that is through internet.^[57]^ With this, it is obvious that it can be a fundamental and unique approach of teletherapy for children with CP specifically throughout coronavirus outbreaks.

Golomb et al^[58]^ carried out experimental research on children diagnosed with paretic CP and revealed that using computer games therapeutic strategies can lead to substantial enhancement in the activity of the hemiplegic upper extremities. Furthermore, Weightman et al^[59]^ and Preston and Ehrsson^[60]^ emphasized that VR is a beneficial technique for increasing motor tasks of upper extremities, and it can be used in both settings, either domestic or at educational institutions. VR applications showed capability for changing manual dexterity among a pediatric population diagnosed with hemiplegic CP.^[61]^ Similarly, Pourazar et al^[62]^ demonstrated substantial betterment among pediatric patients with CP following a VR therapeutic strategy.

### 4.3. Robots for telerehabilitation and management of CP

The use of social assistive robots in TR and management of CP during COVID-19 is supported by this review. Consequently, robots being used for therapeutic purposes makes a better combination with traditional treatment methodologies in the health center, and they hold a higher capacity for regular treatment and guidance in domestic settings by means of streamlined equipment.^[63]^

Taken as a whole, all the studies included in this scoping review reported that TR is beneficial for the management of CP during COVID-19. However, different studies revealed different modes of TR. The majority of the clinical studies focused on the AOT while few also determined the importance of advanced technology such as nonimmersive VR, wearable haptic devices, computer or web-based games, and social assistive robots to be considered as a powerful approach of TR in managing CP during a pandemic.

This review may include some limitations. For instance, this review might not cover every aspect and mode of TR, only populations with CP were considered while other neurological conditions were overlooked. Moreover, the articles included were limited to the English language. Further research is needed on the usage of TR for a variety of neurological and musculoskeletal conditions. Likewise, more specific outcome measures should also be taken into consideration.

## 5. Conclusion

TR has been found to be the safest mode of providing PT to affected children by reducing the stress level among caregivers as well as healthcare practitioners, specifically during COVID-19. Moreover, this innovative and emerging technique also encourages the interest of patients in PT programs. This may also be an engaging and pleasurable mode of rehabilitation specifically for pediatric populations. In the future, it can be an alternate to routine therapy for those who are unlikely to get daily access to in-person therapeutic sessions due to a myriad of reasons or circumstances.

## Author contributions

Conceptualization: Muhammad Kashif, Abdulaziz Albalwi, Syed Abid Mehdi Kazmi.

Data curation: Muhammad Kashif, Syed Abid Mehdi Kazmi, Muhammad Aqeel Aslam.

Formal analysis: Muhammad Kashif, Abdulaziz Albalwi, Syed Abid Mehdi Kazmi, Ahmad A. Alharbi, Kiran Bashir.

Funding acquisition: Muhammad Kashif, Abdulaziz Albalwi, Syed Abid Mehdi Kazmi, Ahmad A. Alharbi, Kiran Bashir, Muhammad Aqeel Aslam, Tamjeed Ghaffar.

Investigation: Muhammad Kashif, Abdulaziz Albalwi, Syed Abid Mehdi Kazmi, Muhammad Aqeel Aslam.

Methodology: Muhammad Kashif, Abdulaziz Albalwi, Muhammad Aqeel Aslam.

Resources: Muhammad Kashif, Ahmad A. Alharbi, Kiran Bashir, Muhammad Aqeel Aslam, Tamjeed Ghaffar.

Validation: Muhammad Kashif.

Visualization: Muhammad Kashif.

Writing—original draft: Muhammad Kashif, Ahmad A. Alharbi.

Writing—review & editing: Muhammad Kashif, Ahmad A. Alharbi.

Project administration: Abdulaziz Albalwi, Syed Abid Mehdi Kazmi, Ahmad A. Alharbi.

Supervision: Abdulaziz Albalwi, Syed Abid Mehdi Kazmi.

Software: Syed Abid Mehdi Kazmi, Kiran Bashir, Tamjeed Ghaffar.
